# Effect of preoperative gabapentin on pain intensity and development of chronic pain after carpal tunnel syndrome surgical treatment in women: randomized, double-blind, placebo-controlled study: Efeito da gabapentina pré-operatória na intensidade da dor e desenvolvimento de dor crônica após o tratamento cirúrgico da síndrome do túnel do carpo em mulheres: estudo randomizado duplo-cego controlado com placebo

**DOI:** 10.1590/1516-3180.2015.00980710

**Published:** 2016-03-18

**Authors:** Eduardo Jun Sadatsune, Plínio da Cunha Leal, Rachel Jorge Dino Cossetti, Rioko Kimiko Sakata

**Affiliations:** I MD, MSc. Anesthesist, Department of Surgery, Universidade Federal de São Paulo (Unifesp), São Paulo, SP, Brazil.; II MD, PhD. Professor, Department of Medicine I, Universidade Federal do Maranhão (UFMA), São Luís, MA, Brazil.; III MD. Professor, Department of Medicine I, Universidade Federal do Maranhão (UFMA), São Luís, MA, Brazil.; IV MD, PhD. Professor, Department of Surgery, Universidade Federal de São Paulo (Unifesp), São Paulo, SP, Brazil.

**Keywords:** Pain, postoperative, Chronic pain, Carpal tunnel syndrome, Neuralgia, Anesthesia, conduction, Dor pós-operatória, Dor crônica, Síndrome do túnel carpal, Neuralgia, Anestesia por condução

## Abstract

**CONTEXT AND OBJECTIVES:**

Effective postoperative analgesia is important for reducing the incidence of chronic pain. This study evaluated the effect of preoperative gabapentin on postoperative analgesia and the incidence of chronic pain among patients undergoing carpal tunnel syndrome surgical treatment.

**DESIGN AND SETTINGS:**

Randomized, double-blind controlled trial, Federal University of São Paulo Pain Clinic.

**METHODS:**

Forty patients aged 18 years or over were randomized into two groups: Gabapentin Group received 600 mg of gabapentin preoperatively, one hour prior to surgery, and Control Group received placebo. All the patients received intravenous regional anesthesia comprising 1% lidocaine. Midazolam was used for sedation if needed. Paracetamol was administered for postoperative analgesia as needed. Codeine was used additionally if the paracetamol was insufficient. The following were evaluated: postoperative pain intensity (over a six-month period), incidence of postoperative neuropathic pain (over a six-month period), need for intraoperative sedation, and use of postoperative paracetamol and codeine. The presence of neuropathic pain was established using the DN4 (Douleur Neuropathique 4) questionnaire. Complex regional pain syndrome was diagnosed using the Budapest questionnaire.

**RESULTS:**

No differences in the need for sedation, control over postoperative pain or incidence of chronic pain syndromes (neuropathic or complex regional pain syndrome) were observed. No differences in postoperative paracetamol and codeine consumption were observed.

**CONCLUSIONS:**

Preoperative gabapentin (600 mg) did not improve postoperative pain control, and did not reduce the incidence of chronic pain among patients undergoing carpal tunnel syndrome surgery.

## RESUMO

**CONTEXTO E OBJETIVOS:**

Analgesia pós-operatória eficaz é importante para reduzir a incidência de dor crônica. Este estudo avaliou o efeito da gabapentina pré-operatória na analgesia pós-operatória e na incidência de dor crônica em pacientes submetidos à cirurgia para tratamento da síndrome do túnel do carpo.

**DESENHO E LOCAL:**

Randomizado, duplo cego, Universidade Federal de São Paulo.

**MÉTODOS:**

Os 40 pacientes com 18 anos ou mais de idade foram distribuídos aleatoriamente em dois grupos: o Grupo Gabapentina recebeu 600 mg de gabapentina no pré-operatório uma hora antes da cirurgia, e o Grupo Controle recebeu placebo. Todos os pacientes receberam anestesia regional intravenosa com lidocaína a 1%. Midazolam foi utilizado para sedação, se necessário. Paracetamol foi administrado para analgesia pós-operatória, conforme necessário, e codeína, se o paracetamol fosse insuficiente. Foram avaliados: a intensidade da dor pós-operatória (durante seis meses), a incidência de dor neuropática pós-operatória (durante seis meses), a necessidade de sedação intra-operatória e o uso de paracetamol e codeína no pós-operatório. A presença de dor neuropática foi estabelecida utilizando-se o questionário DN4 (dor neuropática 4). Síndrome de dor regional complexa foi diagnosticada através do questionário Budapeste.

**RESULTADOS:**

Não foram observadas diferenças na necessidade de sedação, no controle da dor pós-operatória e na incidência de síndromes dolorosas crônicas (neuropáticas ou síndrome de dor regional complexa). Não foram observadas diferenças no consumo de paracetamol e codeína.

**CONCLUSÕES:**

Gabapentina pré-operatória (600 mg) não melhorou o controle da dor pós-operatória e não reduziu a incidência de dor crônica em pacientes submetidos à cirurgia para tratamento da síndrome do túnel do carpo.

## INTRODUCTION

Carpal tunnel syndrome surgery is usually a short outpatient procedure performed under intravenous regional anesthesia (IVRA). However, this type of anesthesia is unable to maintain postoperative analgesia.^1^^,^^2^

Many patients will develop chronic pain after hand surgery, especially complex regional pain syndrome, which is associated with neuropathic, inflammatory and sympathetic dysfunction mechanisms.^3^^,^^4^ Postoperative chronic pain (POCP) has been observed in more than 20% of patients after carpal tunnel release.^1^^,^^5^

The occurrence of postoperative acute pain is an important predictor for the development of chronic pain. Adequate analgesia in the acute postoperative phase reduces the risk of chronic pain. Effective postoperative analgesia is of major importance for preventing POCP.^6^^,^^7^

Gabapentin is known to reduce dorsal horn neuron excitability and central sensitization through various mechanisms.^8^^,^^9^^,^^10^ A review of the literature suggested that the incidence of postoperative chronic pain was reduced through using gabapentin perioperatively.^11^ However, a study on a single preoperative 300 or 600 mg dose of gabapentin prior to caesarean section did not show any improvement in pain control.^12^ Therefore, the role of preoperative gabapentin for postoperative pain control remains a matter of controversy.

This study evaluated the effect of preoperative gabapentin on postoperative pain control and chronic pain incidence among patients undergoing carpal tunnel syndrome surgery under IVRA. The primary objective was to evaluate the effect of preoperative gabapentin on postoperative pain control. The secondary objectives were to investigate the incidence of chronic pain, and the adverse event profile of gabapentin and lidocaine.

## METHODS

Ethics approval for this study was provided by the Ethics Committee of the Federal University of São Paulo under the number 0223/09. The trial was registered at ClinicalTrials.gov (NCT01632215).

### Study design

This was a prospective, randomized, double-blind study.

### Place and setting

All patients underwent surgery performed using the same technique (open carpal tunnel release surgery), by the same medical team at the hospital of the Federal University of São Paulo between 2009 and 2011.

### Participants

The inclusion criteria were that the patients needed to be 18 years of age or older, of either gender, and presenting American Society of Anesthesia (ASA) physical status I or II, before open carpal tunnel release surgery. Carpal tunnel syndrome needed to have been diagnosed by means of clinical examinations (Phalen and Tinel tests) and electromyography (grade ≥ 2). The electromyography scale was graded as follows: normal (grade 0); very mild (grade 1), carpal tunnel syndrome demonstrable only with the most sensitive tests; mild (grade 2), sensory nerve conduction velocity slow on finger/wrist measurement, and normal terminal motor latency; moderate (grade 3), sensory potential preserved with motor slowing, and distal motor latency to abductor pollicis brevis (APB) < 6.5 ms; severe (grade 4), sensory potential absent but motor response preserved, and distal motor latency to APB < 6.5 ms; very severe (grade 5), terminal latency to APB > 6.5 ms; and extremely severe (grade 6), sensory and motor potentials effectively unrecordable (surface motor potential from APB < 0.2 mV in amplitude).^13^

Patients presenting arrhythmia, myocardial ischemia, cognitive impairment, psychiatric disorders, drug abuse, pregnancy, sensitiveness to anesthetics or opioid, anticonvulsant or antidepressant use were excluded.

The sample size was calculated using SPSS 17 for Windows. A reduction of approximately two points or 30% in the numerical rating scale^14^ for pain intensity represented a clinically important difference in chronic pain.^15^ To optimize the relevance of the study findings, a three-point difference in a numerical pain intensity score was chosen to be a clinically meaningful endpoint. For a power of 95% (beta), an alpha level of 5% and an estimated standard deviation of the population of 2.44, based on a preliminary evaluation,^16^ the calculated sample size was 18 patients per group to demonstrate a three-point difference in the scores.

### Randomization, allocation concealment and blinding

The patients were randomly assigned to one of two parallel groups in a 1:1 ratio, through using the computer program Randomizer. Opaque envelopes were prepared in accordance with the computer randomization and were numbered and sealed by a researcher who was not involved in patient assessment. Each envelope contained either a gabapentin or a placebo tablet and was stored at the research hospital, to be given to the research physician prior to each surgery. The gabapentin and placebo tablets were identical in order to maintain patients and researchers blinded to the randomization group. None of the participating physicians or the researchers involved in data collection were aware of the patient study-group randomization. Patients randomized to the Gabapentin Group received 600 mg of gabapentin 1 hour prior to surgery. Patients randomized to the Control Group received placebo.

All patients were asked about dizziness symptoms prior to the onset of anesthesia, and were subsequently assessed for behavioral changes.

### Preoperative and operative procedures

Anesthesia, surgical procedure and patient follow-up were performed by the same research physicians for all patients. Routine monitoring by means of electrocardiogram (ECG), pulse oximetry and noninvasive blood pressure (NIBP) was conducted throughout the duration of patient anesthesia. IVRA was performed with 20 ml of 1% lidocaine, using two tourniquets, in accordance with the technique described by Bier.^2^ In cases of persistent pain after IVRA, local infiltration of 1% lidocaine was performed and the total dose used was recorded. Midazolam was administered for sedation if needed, e.g. if the patient became agitated, and the total dose was recorded.

Paracetamol (maximum of 4 g/24 h) was given if postsurgical analgesia was required, for up to 6 months post-surgery, e.g. if patients reported moderate or severe pain, defined as a numerical scale score ≥ 4 (on a scale ranging from 1 to 10). Codeine (30 mg) was given if paracetamol was insufficient for pain control. The use of other drugs was not allowed for pain control during the follow-up period. The use of specific orthosis was not allowed prior to or after the procedure.

### Outcomes (primary and secondary)

The primary outcome was pain intensity according to a numerical scale (0 = no pain, 10 = worst pain possible) prior to procedure (T-preoperative), at time 0 min (T0 = time of tourniquet release), 30 min, 1 h, 2 h, 2 weeks, 1 month, 3 months and 6 months after the procedure. Secondary outcomes included: total dose of lidocaine supplementation; need for and dose of midazolam; postoperative need for and total dose of paracetamol and/or codeine; development of neuropathic pain and/or complex regional pain syndrome. The DN4 (Douleur Neuropathique 4) questionnaire,^6^ which evaluates seven sensory symptoms and three signs on physical exam, indicated neuropathic pain for patients with a score ≥ 4, measured at different assessment points (before anesthesia and at 2 weeks, 1 month, 3 months and 6 months after surgery). Complex regional pain syndrome was diagnosed using the Budapest questionnaire^15^ on scheduled assessment dates (2 weeks, 1 month, 3 months and 6 months after surgery). Possible side effects were recorded.

### Statistical analysis

SPSS 17 for Windows was used for the statistical analysis. Parametric variables were expressed as means ± standard deviation (SD). Nonparametric variables were described as median, 25^th^ and 75^th^ percentile values. The Mann-Whitney test was used to analyze age, weight, height and body mass index (BMI). The Chi-square test was applied to gender and pain scores. For pain intensity analysis, patients were grouped according to pain intensity score at the different assessment points: mild (score < 4) or moderate/severe pain intensity (score ≥ 4). Proportion tests were used to assess pain intensity score groups between the assessment times (T-preoperative compared with other study points). The McNemar test was used for comparisons within the same study groups. The Mantel-Haenszel test was used for comparisons between study groups. Student’s t test was used to analyze total paracetamol and midazolam doses, anesthesia and surgery duration, and occurrences of complex regional pain syndrome. Fisher’s test was used to evaluate occurrences of neuropathic pain and adverse effects, and the patients’ baseline characteristics (diabetes and prior treatment for carpal tunnel disease). The likelihood test was used to evaluate the electromyography grade of the carpal tunnel syndrome. Missing data were excluded from the analysis.

## RESULTS

**Figure 1.**
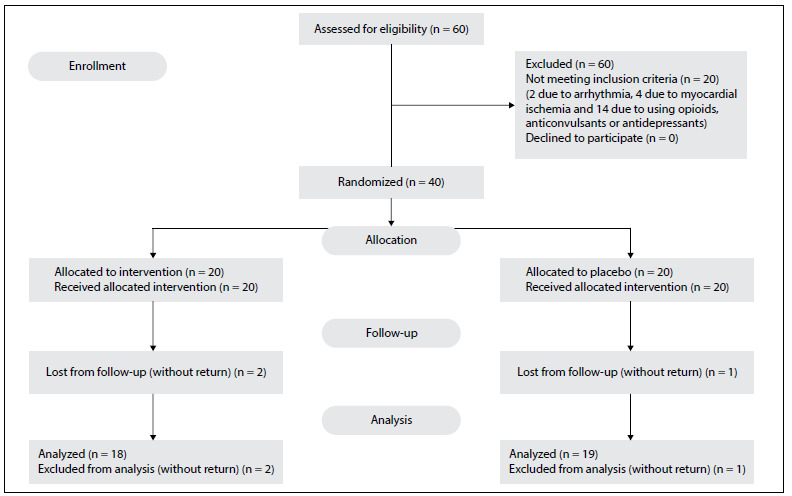
Patients’ recruitment and allocation flowchart.

The patients’ recruitment and allocation flowchart, according to the CONSORT guideline, is shown in Figure 1. Twenty patients were excluded from the study: two due to arrhythmia, four due to myocardial ischemia and 14 due to opioid, anticonvulsant or antidepressant use. A total of 40 patients were included in the study, i.e. 20 in each group.

There were no differences in patient demographics, duration of surgery and anesthesia, need for midazolam use, carpal tunnel syndrome grade or previous diabetic status between the study groups (Table 1). Lidocaine supplementation was not required for any patient. All the patients were ASA I or II, with no difference between the groups (P = 0.642). All the patients were female.

**Table 1.**
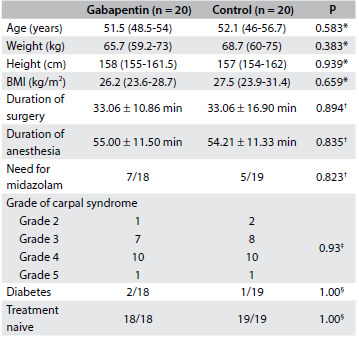
Demographic data of patients with carpal tunnel syndrome

BMI = body mass index. *Mann-Whitney test [median (25^th^ and 75^th^ percentiles)]; ^†^Student’s t test (mean ± SD); ^‡^likelihood test; ^§^Fisher test.

The postoperative assessments showed lower pain intensity scores in comparison with the preoperative baseline pain intensity within the same study groups, except for the T30 min assessment (McNemar’s test, Table 2). However, there was no statistically significant difference in pain intensity scores over time in comparisons across study groups (Mantel-Haenszel test, Table 2). There was no statistically significant difference in the incidence of chronic neuropathic pain (Table 3) or complex regional pain syndrome between the study groups (Table 4).

**Table 2.**
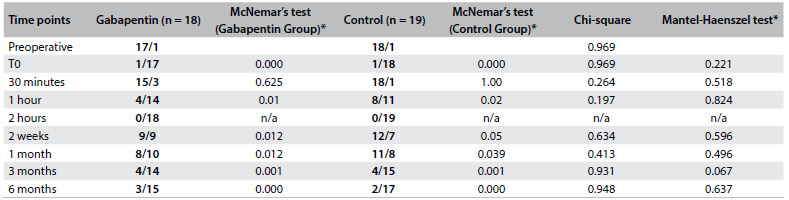
Intensity of pain (pain ≥ 4/pain < 4) among patients operated for carpal tunnel syndrome

T0 = time of tourniquet release; n/a = not applicable; * = P-values.

**Table 3.**
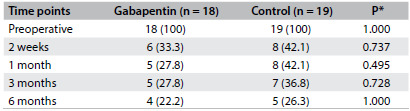
Neuropathic pain (DN4 questionnaire) in patients operated for carpal tunnel syndrome: n (%)

DN4 (Douleur Neuropathique 4) questionnaire; *Fisher’s test.

**Table 4.**
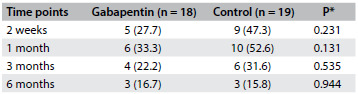
Complex regional pain syndrome according to the Budapest questionnaire among patients operated for carpal tunnel syndrome: n (%)

*Student’s t test.

There was no difference in the use of paracetamol (Gabapentin Group: 24.6 ± 30.1 doses; 95% CI: 13.1 ± 48.1; and Control Group: 30.9 ± 36.9 doses; 95% CI: 9.6 - 39.5; P = 0.572; Student’s t test) or codeine supplementation (Gabapentin Group: 0; Control Group: 1) over the six-month observation period between the study groups.

The adverse effects were similar at the preoperative assessment (beginning of surgery): metallic taste: 4 versus 2 (P = 0.660); oral paresthesia: 2 versus 1 (P = 1.0); dizziness: 4 versus 4 (P = 1.0); somnolence: 4 versus 3 (P = 1.0); and tinnitus 2 versus 4 (P = 0.660); for the Gabapentin and Control groups, respectively. No differences in adverse effects at the end of surgery were noted: metallic taste: 0 versus 1 (P = 1.0); oral paresthesia: 0 versus 0; dizziness: 3 versus 1 (P = 0.605); drowsiness: 2 versus 0 (P = 0.487); and tinnitus: 2 versus 0 (P = 0.486); for the Gabapentin and Control groups, respectively.

## DISCUSSION

The current study did not demonstrate any difference in pain intensity scores, incidence of postoperative chronic pain (POCP) or complex regional pain syndrome (CRPS), need for additional postoperative analgesia or incidence of adverse effects, through using preoperative gabapentin for open carpal tunnel release surgery.

The demographic data were similar between the study groups. Interestingly, only female patients were included, despite no exclusion of male patients. This could be explained by the known higher prevalence of carpal tunnel syndrome in women.^5^

Previous studies have shown that there is a reduced risk of POCP after other types of surgery with the use of gabapentin preoperatively.^17^^,^^18^^,^^19^ In the current report, the observed incidence of POCP and CRPS was lower for the gabapentin group, but it did not reach statistical significance, despite a numerical difference.

Gabapentin was administered one hour prior to surgery to allow for drug absorption.^20^ The use of a single 600 mg dose of gabapentin could explain the lack of efficacy observed. Pre and postoperative gabapentin administration could have prevented central sensitization from previous pain stimulus, surgical trauma and postoperative inflammation, and may have influenced the outcomes.

However, the use of 600 mg of gabapentin preoperatively followed by two-day maintenance had no beneficial effect after total knee arthroplasty.^21^ Also, two study reviews were unable to demonstrate that preoperative gabapentin was effective for prevention of postoperative chronic pain,^22^ even at a higher dose (1200 mg).^23^ However, it should be noted that there was a difference in surgical trauma between those studies and the current report.

A study by Pandey et al.^24^ demonstrated that there was lower opioid consumption and reduced pain scores through using a single pre or postoperative gabapentin dose after open-door nephrectomy, compared with placebo, but no difference between pre or postoperative gabapentin. Further studies are still required, in order to better evaluate the effect of gabapentin on POCP prevention.^25^^,^^26^

The duration of the surgical procedure is known to have an impact on the development of POCP. Procedures that last longer than three hours have been correlated with an increased risk of POCP.^27^ However, the total duration of the surgical procedure in the current study was shorter than three hours, which may have contributed towards reduced incidence of POCP.

Brogly et al.^17^ demonstrated that there was lower incidence of POCP through using preoperative gabapentin prior to thyroidectomy.^17^ The current report only assessed patients until six months of follow-up. However, evaluation after six months would most likely not find any differences in POCP, given that the pain intensity was already mild in both groups at six months.

Surgical procedures that may cause nerve injury have also been correlated with POCP.^1^ Previous studies have reported a wide range of POCP (5 to 80%), which may be related to different definitions in the various studies.^28^^,^^29^ Study design, POCP assessment and interpretation of results may also contribute towards this variability.^28^

The incidence of POCP found in this report was higher than what was previously described.^1^ The observed incidence of POCP was 27.8% and 36.8% at three months, and 22.2% and 26.3% at six months, for G1 and G2, respectively, compared with 21% and 12% at three and six months reported by Yung et al*.*^*1*^ One possible explanation might be the difference in surgical techniques: a limited open carpal tunnel release procedure was used in the above mentioned study. This could result in different efficacy, scar tissue compression of the median nerve and surgery-associated nerve injury, thus leading to POCP.^1^

IVRA is a simple anesthetic technique commonly used for carpal tunnel syndrome release. This type of surgery can usually be performed without intraoperative anesthetic supplementation because IVRA is sufficient for local anesthesia. As expected, there was no need for additional anesthesia in this study. Also, no sedation was required since it is a fast procedure associated with low levels of patient distress.

However, its analgesic effect is limited to the duration of tourniquet use. Additional medication is usually necessary in order to maintain postoperative analgesia,^2^ and this was implemented through using paracetamol and codeine. The use of preoperative gabapentin did not affect postoperative analgesic consumption.

Also, there was no difference in the reported side effects between the groups, as demonstrated by others.^9^^,^^30^ Common lidocaine-related side effects (e.g. metallic taste, oral paresthesia and tinnitus) were also not increased through using gabapentin.

## LIMITATIONS OF THE STUDY

Gabapentin was given as a single preoperative dose, which may have been insufficient to reduce central sensitization and development of POCP. Use of gabapentin and other neuromodulatory drugs was not allowed for treating POCP, which may have contributed towards higher incidence of chronic pain. Additionally, this was a single-center study, which may limit the applicability of our results.

## CONCLUSION

In conclusion, there was no difference in postoperative pain intensity through using a single 600 mg gabapentin dose after open carpal tunnel release surgery.

Further studies are needed in order to determine the best perioperative gabapentin regimen for postoperative pain control and prevention of postoperative chronic pain syndrome after carpal tunnel surgery.
